# Supplementary material for: Therapeutic Potential of Scolopendra subspinipes: A Comprehensive Scoping Review of Its Bioactive Compounds, Preclinical Pharmacology, and Clinical Applications
Source: Toxins (Basel). 2025 May 5;17(5):229. doi: 10.3390/toxins17050229 (PMC12115662; doi:10.3390/toxins17050229)
Supplement: Supplementary file 1 [file toxins-17-00229-s001.zip › toxins-3588068-supplementary.pdf]

Supplementary Table 1. Studies included in the review.

	Authors	Year	Title	Journal
1	**Lee, J.H. et al.**	2015	Anticancer Activity of the Antimicrobial Peptide Scolopendrasin VII Derived from the Centipede, Scolopendra subspinipes mutilans	J Microbiol Biotechnol
2	**Ling, Y. et al.**	2021	ZhiJingSan Inhibits Osteoclastogenesis via Regulating RANKL/NF-κB Signaling Pathway and Ameliorates Bone Erosion in Collagen-Induced Mouse Arthritis	Frontiers in Pharmacology
3	**Wang, Y. et al.**	2017	Centipede venom peptide SsmTX-I with two intramolecular disulfide bonds shows analgesic activities in animal models	J Pept Sci
4	**Yoon, S.Y. et al.**	2018	Pharmacopuncture With Scolopendra subspinipes Suppresses Mechanical Allodynia in Oxaliplatin-Induced Neuropathic Mice and Potentiates Clonidine-induced Anti-allodynia Without Hypotension or Motor Impairment	J Pain
5	**Hua, D. et al.**	2021	Soufeng sanjie formula alleviates collagen-induced arthritis in mice by inhibiting Th17 cell differentiation	Chinese Medicine
6	**Fan, B. et al.**	2025	Soufeng sanjie formula alleviates osteoarthritis by inhibiting macrophage M1 polarization and modulating intestinal metabolites	Journal of Ethnopharmacology
7	**Park, Y.J. et al.**	2018	A novel antimicrobial peptide acting via formyl peptide receptor 2 shows therapeutic effects against rheumatoid arthritis	Sci Rep
8	**Cai, M. et al.**	2013	Scolopendra subspinipes mutilans attenuates neuroinflammation in symptomatic hSOD1(G93A) mice	J Neuroinflammation
9	**Park, Y.J. et al.**	2017	A novel antimicrobial peptide isolated from centipede Scolopendra subspinipes mutilans stimulates neutrophil activity through formyl peptide receptor 2	Biochem Biophys Res Commun
10	**Hong, J.Y. et al.**	2023	Shinbaro2 enhances axonal extension beyond the glial scar for functional recovery in rats with contusive spinal cord injury	Biomedicine and Pharmacotherapy
11	**Hwang, L. et al.**	2018	Scolopendra subspinipes mutilans Extract Suppresses Inflammatory and Neuropathic Pain In Vitro and In Vivo	Evid Based Complement Alternat Med
12	**You, W. et al.**	2023	Scolopendra subspinipes Extracts Protect from Acute Myocardial Infarction via Activating PI3K/Akt signaling	Latin American Journal of Pharmacy
13	**Ali, S.M. et al.**	2019	Biologically active metabolite(s) from haemolymph of red-headed centipede Scolopendra subspinipes possess broad spectrum antibacterial activity	AMB Express

**14**	**Bajpai, V.K. et al.**	2017	Antibacterial Action of Jineol Isolated from Scolopendra subspinipes mutilans against Selected Foodborne Pathogens	Front Microbiol
**15**	**Kim, Y.G. et al.**	2020	Inhibition of Candida albicans and Staphylococcus aureus biofilms by centipede oil and linoleic acid	Biofouling
**16**	**Kwon, Y.N. et al.**	2013	Antimicrobial activity of the synthetic peptide scolopendrasin ii from the centipede Scolopendra subspinipes mutilans	J Microbiol Biotechnol
**17**	**Peng, K. et al.**	2010	Two novel antimicrobial peptides from centipede venoms	Toxicon
**18**	**Lee, J.H. et al.**	2017	Antimicrobial Activity of the Scolopendrasin V Peptide Identified from the Centipede Scolopendra subspinipes mutilans	J Microbiol Biotechnol
**19**	**Lee, W. et al.**	2015	A novel antimicrobial peptide, scolopendin, from Scolopendra subspinipes mutilans and its microbicidal mechanism	Biochimie
**20**	**Hou, H. et al.**	2013	Construction and expression of an antimicrobial peptide scolopin 1 from the centipede venoms of Scolopendra subspinipes mutilans in Escherichia coli using SUMO fusion partner	Protein Expr Purif
**21**	**Chaparro-Aguirre, E. et al.**	2019	Antimicrobial activity and mechanism of action of a novel peptide present in the ecdysis process of centipede Scolopendra subspinipes subspinipes	Sci Rep
**22**	**Lee, B. et al.**	2020	Antibacterial action of lactoferricin B like peptide against Escherichia coli: reactive oxygen species-induced apoptosis-like death	J Appl Microbiol
**23**	**Park, Y.J. et al.**	2015	Antimicrobial peptide scolopendrasin VII, derived from the centipede Scolopendra subspinipes mutilans, stimulates macrophage chemotaxis via formyl peptide receptor 1	BMB Rep
**24**	**Park, Y.J. et al.**	2016	Promotion of formyl peptide receptor 1-mediated neutrophil chemotactic migration by antimicrobial peptides isolated from the centipede Scolopendra subspinipes mutilans	BMB Rep
**25**	**Lee, H. et al.**	2015	Scolopendin 2, a cationic antimicrobial peptide from centipede, and its membrane-active mechanism	Biochim Biophys Acta
**26**	**Choi, H. et al.**	2014	Identification of a novel antimicrobial peptide, scolopendin 1, derived from centipede Scolopendra subspinipes mutilans and its antifungal mechanism	Insect Mol Biol
**27**	**Jo, I.J. et al.**	2013	Scolopendra subspinipes mutilans protected the cerulein-induced acute pancreatitis by inhibiting high-mobility group box protein-1	World J Gastroenterol
**28**	**Yoo, W.G. et al.**	2014	Antimicrobial peptides in the centipede Scolopendra subspinipes mutilans	Funct Integr Genomics
**29**	**Choi, H. et al.**	2013	Antifungal effect and pore-forming action of lactoferricin B like peptide derived from centipede Scolopendra subspinipes mutilans	Biochim Biophys Acta

**30**	**Kim, S. et al.**	2020	Lactoferricin B like peptide triggers mitochondrial disruption-mediated apoptosis by inhibiting respiration under nitric oxide accumulation in Candida albicans	IUBMB Life
**31**	**Lee, H. et al.**	2016	Scolopendin 2 leads to cellular stress response in Candida albicans	Apoptosis
**32**	**Lee, H. et al.**	2017	Scolopendin, an antimicrobial peptide from centipede, attenuates mitochondrial functions and triggers apoptosis in Candida albicans	Biochem J
**33**	**Yoon, M.A. et al.**	2006	Antioxidant effects of quinoline alkaloids and 2,4-di-tert-butylphenol isolated from Scolopendra subspinipes	Biol Pharm Bull
**34**	**Alam, M.B. et al.**	2017	Inhibition of melanogenesis by jineol from Scolopendra subspinipes mutilans via MAP-Kinase mediated MITF downregulation and the proteasomal degradation of tyrosinase	Sci Rep
**35**	**Kong, Y. et al.**	2013	A Novel Factor Xa-Inhibiting Peptide from Centipedes Venom	Int J Pept Res Ther
**36**	**Lee, W. et al.**	2016	Antithrombotic and antiplatelet activities of small-molecule alkaloids from Scolopendra subspinipes mutilans	Sci Rep
**37**	**Kong, Y. et al.**	2013	Purification and characterization of a novel antithrombotic peptide from Scolopendra subspinipes mutilans	J Ethnopharmacol
**38**	**Ding, D. et al.**	2016	Two new isoquinoline alkaloids from Scolopendra subspinipes mutilans induce cell cycle arrest and apoptosis in human glioma cancer U87 cells	Fitoterapia
**39**	**Guo, Y.R. et al.**	2017	A New 1,5-Dihydroxy-4-methoxyisoquinoline from Scolopendra subspinipes mutilans	Chem Biodivers
**40**	**Ma, W. et al.**	2014	Extracts of centipede Scolopendra subspinipes mutilans induce cell cycle arrest and apoptosis in A375 human melanoma cells	Oncol Lett
**41**	**Hu, Y.X. et al.**	2023	Antihepatoma peptide, scolopentide, derived from the centipede scolopendra subspinipes mutilans	World Journal of Gastroenterology
**42**	**Zhao, H. et al.**	2012	Antitumor and immunostimulatory activity of a polysaccharide-protein complex from Scolopendra subspinipes mutilans L. Koch in tumor-bearing mice	Food Chem Toxicol
**43**	**Mao, Q.Y. et al.**	2024	Scorpiones, Scolopendra and Gekko Inhibit Lung Cancer Growth and Metastasis by Ameliorating Hypoxic Tumor Microenvironment via PI3K/AKT/mTOR/HIF-1α Signaling Pathway	Chinese Journal of Integrative Medicine
**44**	**Hu, B.Y. et al.**	2024	Structurally Diverse Alkaloids with Anti-Renal-Fibrosis Activity from the Centipede Scolopendra subspinipes mutilans	Journal of Natural Products
**45**	**Ren, Y. et al.**	2006	Relevant activities of extracts and constituents of animals used in traditional Chinese medicine for central nervous system effects associated with Alzheimer's disease	J Pharm Pharmacol

**46**	**Seo, Y.S. et al.**	2019	Protective Effects of Scolopendra Water Extract on Trimethyltin-Induced Hippocampal Neurodegeneration and Seizures in Mice	Brain Sci
